# Treatment of Latent Tuberculosis Infection

**DOI:** 10.1007/s40506-017-0135-7

**Published:** 2017-09-22

**Authors:** Patrick Tang, James Johnston

**Affiliations:** 1 Department of Pathology, Sidra Medical and Research Center, P.O. Box 26999, Doha, Qatar; 2 British Columbia Centre for Disease Control, Vancouver, Canada

**Keywords:** Latent tuberculosis infection, Chemoprophylaxis, Isoniazid, Rifampin, Interferon-gamma release assay, Tuberculin skin test

## Abstract

The treatment of latent tuberculosis infection (LTBI) is an essential component of tuberculosis (TB) elimination in regions that have a low incidence of TB. However, the decision to treat individuals with LTBI must consider the limitations of current diagnostic tests for LTBI, the risk of developing active TB disease, the potential adverse effects from chemoprophylactic therapy, and the importance of treatment adherence. When an individual has been diagnosed with LTBI and active TB has been ruled out, this is followed by an evaluation of the risks and benefits of LTBI treatment within the context of the regional epidemiology of TB and public health priorities. Once the decision to treat LTBI has been reached, and the infection is not suspected to be due to drug-resistant TB, the recommended regimens include isoniazid and/or rifamycin-derivatives, and the choice of regimen will depend upon the clinical considerations for that individual, such as patient preference, concomitant medications, hepatic disease, pregnancy, or immunodeficiency. As the duration of treatment of LTBI therapy is many months, therapy must be offered within a plan that monitors for adverse drug reactions and emphasizes adherence. For latent multidrug-resistant TB (MDR-TB) or extensively drug-resistant TB (XDR-TB) infection, the management is more complicated as there are few options for chemoprophylactic therapy and little evidence regarding the efficacy or risks of these regimens.

## Introduction

Tuberculosis is a leading cause of death worldwide [1]. The causative organism is *Mycobacterium tuberculosis*, and for most infected individuals, the organism establishes a latent TB infection (LTBI), where the mycobacteria remain viable within the host but the individual is asymptomatic and non-infectious. For the majority of LTBI cases, the *M. tuberculosis* remains latent for the lifetime of the host. However, if the host immune system is unable to contain the mycobacteria, the organism will have an opportunity to progress to an active, symptomatic infection in the host. The risk of developing active TB following LTBI is dependent on many factors, especially the immunological status of the infected host [2••]. If effective chemoprophylaxis is given to individuals with LTBI, the risk of developing active TB is significantly reduced [3].

The feasibility and public health impact of LTBI treatment depend in part on the epidemiology and health care resources of a region [4••]. In high resource, low TB incidence settings, identification, and treatment of LTBI cases are an important part of the public health strategy for TB prevention and elimination. In regions where the TB incidence is high, the feasibility and public health impact of LTBI therapy are less clear.

Diagnostic testing and clinical evaluation for LTBI are typically targeted to individuals who are at high risk of acquiring *M. tuberculosis* infection or at high risk of developing active TB disease following infection with *M. tuberculosis* [2••]. Testing includes a clinical and radiological evaluation which can rule out active TB disease and determine risk of progression to active TB disease [5••]. In the absence of active TB disease, immunologic testing for LTBI is also required. Generally, there are two types of immunologic tests available for the diagnosis of LTBI: the Mantoux tuberculin skin test (TST) which measures a delayed-type hypersensitivity reaction following intradermal inoculation with *M. tuberculosis* antigens [6], and interferon-gamma release assays (IGRAs), which measure the release of interferon-gamma from T cells in blood samples exposed to *M. tuberculosis*-specific peptides [7]. The two commercially available IGRAs are US Food and Drug Administration-approved: QuantiFERON-TB Gold and T-SPOT.TB. A positive TST or IGRA can be an indication of infection with *M. tuberculosis*, especially when the history indicates a risk of exposure to *M. tuberculosis*. The value of these tests is greatest when they are performed with the intent to treat all cases of LTBI that are identified, and thus, they must be part of a program that includes patient counseling, treatment, and follow-up [8, 9].

For the treatment of LTBI where the source is unlikely to be drug-resistant *M. tuberculosis*, there are four recommended regimens for therapy and there is considerable evidence for the risks and benefits of treatment: (1) isoniazid daily or semi-weekly for 9 months, (2) isoniazid daily or semi-weekly for 6 months, (3) isoniazid and rifapentine weekly for 3 months, or (4) rifampin daily for 4 months [2••, 10]. There are special considerations when choosing a regimen such as HIV infection, pregnancy, age, hepatic disease, and the potential to interact with other medications. Because of the long duration of therapy and potential for adverse effects from these medications, patients must be monitored for adherence and adverse events (e.g., liver toxicity from isoniazid [11], rash from rifampin [12], etc.).

When the source of the LTBI is suspected to be drug-resistant *M. tuberculosis*, there is less evidence to guide the optimal regimen for LTBI treatment [13, 14]. Because of the lack of evidence and significant potential for adverse effects, the first decision is whether to closely follow the patient with LTBI to ensure early detection and treatment if active disease were to develop or to treat the LTBI. The considerations for treatment of LTBI from drug-resistant TB include the individual risk for progression to active disease, the antibiotic susceptibility profile of the source organism and the individual’s risk for adverse drug reactions from the proposed therapy.

## Diagnosis of LTBI

The decision to treat LTBI must balance the individual and public health risks of TB disease against the potential risks of preventative therapy. Certain risk factors can elevate one’s risk for developing active TB including HIV infection, recent exposure (< 2 years) to an active, infectious TB case, fibrotic changes on chest X-ray consistent with past TB infection, silicosis, organ transplantation, immune suppressing medications (such as tumor necrosis factor inhibitors and high-dose glucocorticoid therapy), end stage kidney disease requiring dialysis, and carcinomas of the head and neck. When an individual has a positive TST or IGRA and one of these risk factors, treatment for LTBI is strongly recommended [3]. Other risk groups include injection drug users, homeless, and incarcerated individuals. It is also recommended that individuals in these risk groups be screened for LTBI and offered treatment if they have a positive TST or IGRA [15]. The evidence is not as clear for screening other high risk groups, such as recent immigrants from high-incidence countries, people with diabetes, health care workers, and people with an alcohol abuse history, and this will be dependent on local resources and epidemiology.

The TST using the Mantoux technique has been in use for over 100 years [6]. A standardized dose of 5 tuberculin units (TU) of *M. tuberculosis* purified protein derivative (PPD) is injected intradermally, and the delayed hypersensitivity response is assessed 48 to 72 h later. A positive TST reaction is based on both the diameter of the TST induration and the clinical risk factors of the patient [8]. The TST (and IGRAs) does not distinguish between latent and active TB infection, so active TB infection must be ruled out in those who have a positive test result [4••].

An induration of 5 mm or more is considered positive in high-risk individuals with the following risk factors: HIV infection, recent contact with an active TB case within the past 2 years; fibronodular changes on chest X-ray consistent with healed TB (but not previously treated); immune suppression (e.g., from organ transplantation, TNF-α inhibitors, corticosteroids, etc.); or end-stage kidney disease requiring dialysis.

An induration of 10 mm or more is considered positive in individuals who are as follows: recent migrants from countries with high TB prevalence; injection drug users; residents, and staff in high-risk congregate settings (e.g., prisons, long-term care facilities, hospitals, homeless shelters, etc.); mycobacteriology laboratory staff; persons with clinical conditions which elevate TB risk such as diabetes, malnutrition (low body weight), cigarette smoking, daily alcohol consumption (> 3 drinks/day), silicosis, hematologic malignancies, and certain carcinomas (e.g., the head and neck); or children exposed to adults in high-risk categories.

False negative TST reactions can occur in young children (< 5 years), early infections (< 8 weeks post-infection), individuals who have recently received vaccinations, immunocompromised individuals (such as HIV infection or treatment with immunosuppressive drugs), and those who have disseminated TB infection. False-positive TST reactions can occur in individuals who have had BCG vaccinations or infection with non-tuberculous mycobacteria [2••].

Interferon-gamma release assays can be used in place of or in conjunction with the TST to support the diagnosis of LTBI. In contrast to the TST, where a mixture of many TB antigens is inoculated into the patient, IGRAs employ only two or three *M. tuberculosis*-specific antigens in an in vitro blood test; these antigens are not shared with the *M. bovis* BCG vaccine strain and most non-tuberculous mycobacteria (except for *M. marinum*, *M. kansasii*, *M. szulgai*, *and M. flavescens*) [16•]. As a result, IGRAs appear to be more specific than the TST. Blood from the patient is incubated with the TB-specific antigens, and the release of interferon-γ from the T cells is measured. An increased level of interferon-γ release is indicative of infection with *M. tuberculosis*.

The most commonly used commercial IGRAs are the QuantiFERON-TB Gold and the T-SPOT.TB assays [17]. The QuantiFERON-TB Gold test employs three TB-specific antigens (ESAT-6, CFP-10, and TB7.7) which are incubated with a whole blood sample from the patient. The production of interferon-γ by the T cells in response to the TB antigens is measured using ELISA [16•]. The QuantiFERON-TB Gold test is positive when the difference in interferon-γ concentration between the TB antigens and the nil control is greater than or equal to 0.35 IU/mL. In contrast to the QuantiFERON-TB Gold test, the T-SPOT.TB assay employs two TB-specific antigens (ESAT-6 and CFP-10) and requires the isolation of peripheral blood mononuclear cells (PBMCs) from the blood sample [16•]. Interferon-γ produced by each cell is detected in a sandwich ELISA technique known as the enzyme-linked immunospot (ELISPOT) assay. Each cell that produced interferon-γ would create a spot on the microtiter well. If the negative well has less than or equal to five spots and either of the antigen wells has more than six spots greater than the nil control, it is considered positive. One of the concerns regarding the use of IGRAs is reproducibility, especially when employed in serial screening of health care workers [18]. Until more experience with these tests is accumulated and more nuanced interpretation guidelines available, IGRA results that are near the manufacturer-recommended cutoff points must be interpreted with caution [19].

## Treatment regimens for LTBI

There are four potential drug regimens recommended for the treatment of LTBI (Table 1). These regimens are recommended only for the treatment of LTBI when the source organism is not suspected to be drug resistant. The 9-month isoniazid regimen is supported by the most evidence [2••]. Although individuals who complete 9 months of isoniazid have lower rates of reactivation compared to 6 months [20], when compliance, cost, and hepatotoxicity are considered, the 6-month course of isoniazid is an acceptable alternative [21]. The efficacy and safety of the 6-month isoniazid and various other regimens, including ones with rifamycins, have been systematically reviewed [22–24]. In terms of efficacy at preventing the reactivation of TB, no regimen showed superiority over the others. In terms of safety, the 4-month rifampin and 3-month rifapentine plus isoniazid regimens appear to have fewer hepatotoxicity events compared to the longer isoniazid-only regimens. The shorter duration regimens are often preferred by patients and result in greater compliance rates [20].

**Table 1:** Treatment regimens for LTBI

Isoniazid—9 months	
*Isoniazid*:	
Standard dosage	Adults 5 mg/kg/day (up to 300 mg/day) p.o. for 9 months or 900 mg twice weekly p.o. for 9 months
	Children 10–15 mg/kg/day (up to 300 mg/day) p.o. for 9 months or DOT 20–30 mg/kg (up to 900 mg) twice weekly p.o. for 9 months
Contraindications	Caution in people with hepatic impairment; contraindicated in people with acute hepatic disease, or prior isoniazid hepatic injury
Main drug interactions	Increases serum phenytoin, carbamazepine. Increases hepatotoxicity when combined with rifampin, pyrazinamide, ethanol, and acetaminophen
Main side effects	Asymptomatic hepatic aminotransferase elevation, clinical symptomatic hepatitis, and peripheral neuropathy
Isoniazid—6 months	
*Isoniazid*:	
Standard dosage	Adults 5 mg/kg/day (up to 300 mg/day) p.o. for 6 months or 900 mg twice weekly × 6 months
	Children 10–15 mg/kg/day (up to 300 mg/day) p.o. for 6 months or DOT 20–30 mg/kg (up to 900 mg) twice weekly for × 6 months
Contraindications	Caution in people with hepatic impairment; contraindicated in people with acute hepatic disease, or prior isoniazid hepatic injury
Main drug interactions	Increases serum phenytoin, carbamazepine. Increases hepatotoxicity with rifampin, pyrazinamide, alcohol, and acetaminophen
Main side effects	Asymptomatic hepatic aminotransferase elevation, clinical symptomatic hepatitis, and peripheral neuropathy
Isoniazid and Rifapentine—12 weeks	
*Isoniazid*:	
Standard dosage	Age 2–11: 25 mg/kg rounded to nearest 50 or 100 mg with a maximum 900 mg p.o. weekly (DOT); over age 12: 15 mg/kg rounded to nearest 50 or 100 mg maximum 900 mg p.o. weekly (DOT)
Contraindications	Caution in people with hepatic impairment; contraindicated in people with acute hepatic disease or prior isoniazid hepatic injury
Main drug interactions	Increases serum phenytoin, carbamazepine. Increases hepatotoxicity with rifampin, pyrazinamide, alcohol, and acetaminophen
Main side effects	Asymptomatic hepatic aminotransferase elevation, clinical symptomatic hepatitis, and peripheral neuropathy
*Rifapentine*:	
Standard dosage	Weight **>** 50 kg 900 mg; 32.1–50 kg 750 mg; 25.1–32 kg 600 mg; 14.1–25 kg 450 mg; 10–14 kg 300 mg. All given p.o. weekly under DOT
Contraindications	Prior hypersensitivity reaction; caution in HIV infected individuals with careful attention paid to drug-drug interactions with antiretrovirals
Main drug interactions	Warfarin, methadone, anticonvulsants, and multiple antiretrovirals.
Main side effects	Rash, orange discoloration of body fluids, hepatotoxicity, and hypersensitivity reaction, ranging from flu-like syndrome to anaphylaxis (3.5%). Nausea, vomiting, and hepatotoxicity
Rifampin—4 months	
*Rifampin*:	
Standard dosage	10 mg/kg/day (up to 600 mg) p.o. daily for 4 months
Contraindications	Prior hypersensitivity reactions; caution in HIV infected individuals with careful attention paid to drug-drug interactions with antiretrovirals
Main drug interactions	Warfarin, methadone, anticonvulsants, and multiple antiretrovirals
Main side effects	Rash, orange discoloration of body fluids, hematologic syndromes, flu-like illness, and hepatotoxicity

Four drug regimens recommended for the treatment of LTBI

For clinicians and public health teams overseeing LTBI treatment programs, regimens that incur additional resource requirements also be considered such as the need for directly observed therapy (DOT) for the weekly dosing regimens and the higher cost of rifapentine. As such, when there are no clinical factors restricting the choice of treatment regimens, the choice must also take into account resource capacities of each LTBI program. For individuals living with HIV who are taking anti-retroviral drugs, the isoniazid-only regimens are often chosen to avoid drug interactions between rifamycins and anti-retroviral therapy, and the 9-month course is considered more efficacious than 6 months [25•]. For children, all the four recommended regimens have been shown to have similar efficacies and rates of adverse effects [26] with the caveat that the isoniazid plus rifapentine regimen should not be used in children under 2 years old. The isoniazid-rifapentine regimen is also currently not recommended in pregnant women and women expecting to be pregnant during the treatment period but there are ongoing clinical trials to evaluate the pharmacokinetics and safety of this regimen in pregnancy (IMPAACT P2001). Finally, regimens containing pyrazinamide are no longer recommended due to the increased risk of hepatotoxicity and death [27].

It is essential to monitor for adverse drug reactions during treatment. For individuals taking self-administered therapy, monthly or more frequent assessments by a health care provider is required [28••]. The potential reactions from isoniazid include peripheral neuropathy and effects on liver function from asymptomatic elevations in liver enzymes to severe hepatotoxicity [21, 29, 30]. Rifamycin-containing regimens may lead to cutaneous reactions, hypersensitivity, gastrointestinal intolerance, hematological syndromes, and hepatotoxicity [31, 32]. In addition, patients must be advised that rifampin and rifapentine will cause an orange discoloration of body fluids and that contact lenses may become stained [33]. It is important to elicit the list of current medications from the patient to determine potential drug interactions with isoniazid and rifamycins. For example, isoniazid can increase levels of phenytoin and carbamazepine, and rifamycins can decrease levels of many drugs including oral contraceptives, warfarin, and sulfonylureas [34]. Patients must also be educated to contact their health care provider if certain symptoms develop such as abdominal pain, anorexia, nausea, vomiting, dark urine, pale stools, jaundice, or persistent weakness [2••].

The indications for performing routine baseline laboratory testing for serum aspartate aminotransferase, alanine aminotransferase, and bilirubin can vary between different institutions and regions. Generally, baseline liver function tests are indicated for isoniazid-containing regimens in the presence of risk factors for hepatic toxicity, such as age > 35 years, pre-existing liver disease, regular alcohol use, HIV infection, pregnancy, or within 3 months postpartum [2••, 28••]. These tests are typically performed at the monthly assessments and the frequency of testing dependent on age and risk factors.

## Treatment for contacts of MDR-TB

There have been few studies to determine the effectiveness of LTBI treatment for contacts of drug-resistant TB cases [13, 14]. Existing studies also use a wide range of treatment regimens in different patient populations, making the comparison of the efficacy of different regimens difficult [35•]. Fluoroquinolone-based treatment regimens appear most promising such as a fluoroquinolone alone or in combination with ethambutol [36, 37•, 38], but no regimen has been fully evaluated in a randomized control trial setting. At present, there are at least three ongoing randomized trials (VQUIN, TB-CHAMP, PHOENIx) evaluating LTBI treatment in MDR-TB contacts. Results, however, will not be available for several years [39].

The decision to treat LTBI in an individual exposed to an MDR-TB source case must be based on individual-risk assessments for each patient and the drug susceptibility profile of the suspected source organism. This treatment program must also take into account the quality of the drug susceptibility results, prior exposure of the patient to active TB; the resources required to follow the patient during treatment and monitor for adverse drug reactions, as well as the potential for inducing acquired drug resistance [40]. The cost of treatment and diversion of resources from the treatment of active MDR-TB cases must also be taken into consideration [4••]. In some cases and settings, clinical and/or periodic radiologic examinations for at least 2 years may be preferable to prophylactic treatment for contacts of MDR-TB cases [13].

The quality of evidence to support the treatment of contacts of MDR-TB still remains low. Current treatment guidelines for MDR-TB contacts with LTBI are based on expert opinion and observational studies only [13]. In a recent systematic review and meta-analysis evaluating observational studies on LTBI therapy in MDR-TB contacts, the authors found that MDR-TB contacts treated for LTBI had a reduced incidence of active TB, suggesting that treatment may be effective in preventing progression to MDR-TB [35•]. In particular, fluoroquinolone-based regimens (with or without ethambutol) ranging from 6 to 12 months in duration appear to be most promising. In contrast, however, pyrazinamide-based regimens have high rates of discontinuation related to adverse effects. LTBI therapy in MDR-TB contacts should be considered in consultation with a physician with expertise in MDR-TB therapy. Alternatively, given the paucity of evidence to support LTBI treatment in MDR-TB contacts, close clinical and radiological follow-up could be considered a viable alternative especially in regions with low TB incidence [41].

## Conclusions

Although the management of active TB cases is first and foremost for all TB control programs, the goal of TB elimination also requires the identification and treatment of LTBI. Diagnostic tests for LTBI are most effective when targeted at individuals who are at high risk for acquiring TB infection or at high risk for reactivation of LTBI. Chemoprophylactic treatment of individuals with LTBI must balance the risks and benefits of treatment for these asymptomatic individuals as the various regimens use drugs which have the potential for serious adverse effects. The regimen backed by the most evidence is 9 months of isoniazid therapy. However, with considerations for adverse effects, patient uptake and adherence, alternative regimens with shorter duration and/or with rifamycins are also effective for reducing the likelihood of TB reactivation. For individuals exposed to cases of MDR-TB, there is currently little evidence to recommend specific drug regimens but ongoing randomized control trials should yield new recommendations in the upcoming years.
